# Estimation of the Number of Quantum Dots Immobilized on an Ultra-flat Au Surface

**DOI:** 10.1186/s11671-017-2056-2

**Published:** 2017-04-26

**Authors:** Hiroki Ito, Atsushi Iio, Katsutoshi Tokuhara, Hiroyuki Sakaue, Yutaka Kadoya, Hitoshi Suzuki

**Affiliations:** Graduate School of Advanced Sciences of Matter, Hiroshima University, 1-3-1 Kagamiyama, Higashi-hiroshima, 739-8530 Japan

**Keywords:** Quantum dot, Immobilization, Self-assembled monolayer, Atomic force microscopy

## Abstract

Quantum dots (QDs) were immobilized on an ultra-flat Au surface by using amide binding between the carboxyl groups on the QDs and the amino groups of the self-assembled monolayer on the surface. The number density of the QDs estimated by atomic force microscopy (AFM) agreed with the quantity of QDs estimated by X-ray photoelectron spectroscopy and fluorescence microscopy. QDs were also immobilized on dot patterns fabricated by e-beam lithography. AFM was able to identify clusters of just a few QDs on the dot patterns, whose minimum designed size was 50 nm × 50 nm per dot.

## Background

Colloidal quantum dots (QDs), which are nanometer-scale semiconductor particles suspended in a solvent, are prospective materials for highly efficient electronic and optoelectronic devices, such as field electric transistors, photovoltaics, and light-emitting diodes [1–3]. To fabricate such devices, techniques to immobilize QDs on the designed areas of the devices are required. In addition, control of the number of QDs immobilized on the designed areas, especially for nanostructures, is another important factor in constructing QD-based devices. For example, for single-electron devices, a single or a few QDs, which serve as a Coulomb island, must be fixed between two electrodes [4]. An example of an optical application was the confinement of the propagating direction of light by an optical Yagi-Uda antenna, which was driven by a single QD fixed on the feed element [5–9]. Propagation of a plasmon polariton on a metal nanowire was also demonstrated by a single QD coupled with the nanowire [10, 11].

The formation of a self-assembled monolayer (SAM) is a popular and effective method to generate an appropriate surface structure for binding QDs to the surface. On Au surfaces, a suitable choice for monolayer formation is alkanethiols, whose S atom specifically binds to surface Au atoms. This allows amino-terminated Au surfaces to be fabricated by using amino-alkanethiol molecules [12–15]. The amino groups on the modified Au surface can then bind with carboxyl groups on the QDs by forming amide bonds [16]. In this case, the SAM serves as a binder between the QDs and the surface.

However, analysis of the number of QDs fixed on the surface is technically challenging. The quantities of the QDs on the surface can be estimated based on the intensity of fluorescence from the QDs or the atomic composition of the QDs obtained by X-ray photoelectron spectroscopy (XPS). These methods, however, do not give the exact number of QDs nor have enough spatial resolution to identify a single QD. It was found by fluorescence microscopy that a single QD showed blinking behaviors of its fluorescence [17–19]. However, the exact QD number density on the surface and the distance between two QDs are difficult to measure without advanced techniques such as spectral imaging in optical microscopy [20]. In principle, scanning electron microscopy (SEM) and atomic force microscopy (AFM) have sufficient spatial resolution to identify an individual QD on the surface [4, 21–23]. However, the imaging of surface-bound QDs by SEM is frequently prevented by surface organic components, including contaminants, and by denaturation of the SAM. Meanwhile, the observation of QDs by AFM depends on the roughness of the substrate; hence, a single QD on a rough surface, such as an Au thin film prepared by thermal evaporation, is difficult to distinguish from a surface protrusion, because such protrusions are comparable in size to the QD.

In this study, we immobilized QDs on an ultra-flat Au surface containing an SAM. The number of QDs was exactly counted from AFM images and compared with the quantity of QDs estimated by XPS and fluorescence microscopy. QDs were also fixed on patterns that had been fabricated by e-beam lithography, and the number of the QDs on the patterns was analyzed by AFM.

## Methods

### Preparation of an Ultra-flat Surface

An Au substrate having an ultra-flat surface was prepared according to the previously reported method [24–26], as follows. An Au film was deposited by vacuum evaporation on a glass slide that had been cleaned with piranha solution and dried by N_2_ gas blowing. The thickness of the film was approximately 600 nm. A glass substrate was attached to the Au surface using an epoxy adhesive (Norland, Optical Adhesive 61), and the Au film was then peeled off from the glass slide with tweezers. The Au surface that had been attached to the glass slide was used as the ultra-flat Au surface. The ultra-flat Au surface was cleaned by UV/O_3_ exposure before formation of SAM.

### Immobilization of QDs with SAM

A self-assembled monolayer having amino groups on the ultra-flat Au surface was formed by immersing the substrate in an 1 mM ethanol solution of 11-amino-1-undecanethiol (Dojindo laboratories) at 35 °C for 24 h and rinsing the substrate with ethanol, ultra-pure water, and borate buffer solution (pH 9.1). The QDs used in this study had carboxyl groups on their surfaces (Invitrogen, Qdot 800 ITK carboxyl). The height of QD on a glass slide measured approximately 8 nm by AFM. The SAM-covered substrate was immersed in a borate buffer solution of the QDs, including water-soluble carbodiimide (Dojindo) (5 mM), at 35 °C for 24 h to bind the carboxyl groups on the QDs to the amino groups on the Au surface by the amide binding. After that, the substrate was rinsed with borate buffer solution and ultra-pure water and dried by N_2_ gas blowing.

### Immobilization of QDs on a Patterned Surface

Resist patterns on the ultra-flat Au substrate were fabricated by e-beam lithography. The resist patterns consisted of arrays of dots of bare Au surface, with the remaining area covered by the resist (ZEP520, ZEON). The patterns were designed with square dot having sides 50 nm, 100 nm, 500 nm, 1 *μ*m, or 10 *μ*m. The thickness of the resist was approximately 80 nm. An amino-terminated SAM was formed on the bare dots, and the QDs were immobilized by the same method as described above. Then, the resist covering the surface (except on the bare dots) was removed by solvent. The detailed process is described in [27].

### Estimation of the Number of QDs

The quantity of QDs on the substrate was estimated using XPS. The QDs used in this study consisted of a CdSe core with a ZnS shell, covered with a polymer containing carboxyl groups. The atomic ratio of Cd thus reflects the quantity of QDs fixed on the surface. The XPS measurements were conducted with a PerkinElmer PHI 1600 ESCA system. The X-ray source was Mg K _*α*_ (1254.6 eV). For charge correction of the binding energy, the hydrocarbon component was adjusted to 284.6 eV in the C _1s_ peak.

Fluorescence from the QDs was observed with a fluorescence microscope (BX50, Olympus) with an objective lens (×10, numerical aperture (NA) 0.4), a filter set consisting of an emission filter (D800/50, Chroma), an excitation filter and a dichroic mirror (D605B, Semrock), and an electron-multiplying CCD (EMCCD) camera (iXon 897, Andor). The gain of the EMCCD camera was set to a constant value (800), and the measured intensity was linear. Using the above lens with low NA and low magnification, the obtained images did not have high enough sensitivity to detect the fluorescence of a single QD.

AFM observation was carried out in ambient conditions with a Nanoscope IIIa (Digital Instruments).

## Results and Discussion

### Density of the Immobilized QDs

**Fig. 1:**
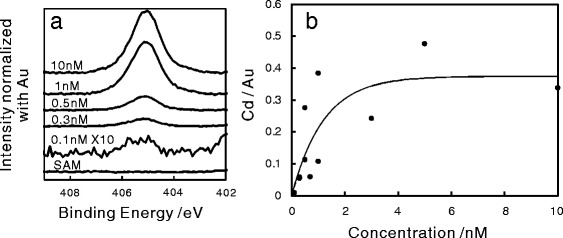
**a** XPS spectra of the substrates with/without QDs. **b** Atomic ratio of Cd/Au as a function of the concentration of QD solution

The XPS spectra of the Au surfaces with/without QDs are shown in Fig. 1 a. The substrate covered with the SAM, but not exposed to the Cd solution, displayed no XPS peak for Cd, whereas those immersed in the QD solution did contain Cd; this element is one of the main components of the QDs (Fig. 1 a). Inspection of the spectra suggests that the amount of QDs on the surface depended on the concentration of QDs in the solutions in which the substrates were immersed.

The atomic ratio of Cd/Au, an indicator of the amount of QDs immobilized on the Au surface, increased with the concentration of the QDs (Fig. 1 b). This confirms that the amount of the QDs fixed on the Au surface increased with the concentration of the QDs in the solution. The trend of the XPS data can be interpreted by a fitting curve based on Langmuir isotherm adsorption model [28]. Each XPS datum was obtained from a measurement on a single spot, which was an approximately circular area with a radius of 0.4 *μ*m. The deviation of the data from the fitting curve indicates a degree of spatial inhomogeneity in the amount of QDs on the surface. Nonetheless, the analysis based on the Langmuir model indicates that the QDs were immobilized on the SAM, forming a monolayer but not a multilayer.

**Fig. 2:**
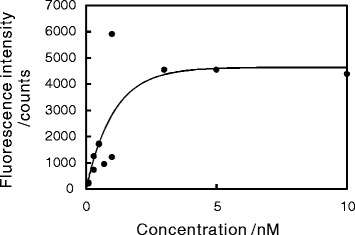
Fluorescence intensity as a function of the concentration of QD solution

The measured fluorescence intensities of the QDs further confirm that the amount of QDs on the surface depended on the concentration of QDs in the solution (Fig. 2). The fluorescence intensities, in units of counts/pixel, were the mean value obtained from nine images taken with the EMCCD camera. Like the XPS data, the fluorescence intensities were also consistent with the Langmuir isotherm adsorption model. In general, the fluorescence of a QD on a metal surface depends on the distance between the QD and the surface, because of both the quenching and enhancement of fluorescence [29]. In this study, the distance between the QDs and the Au surface was in principle determined by the thickness of the SAM and that of the polymer covering the QDs. The observation of fluorescence of the QDs suggests that the QDs were separated at a moderate distance from the Au surface by the SAM and the polymer. However, the total intensity was relatively weaker, because the distance between the QDs and the metal surface was not optimal for emission, and fluorescence of some of the QDs were quenched.

**Fig. 3:**
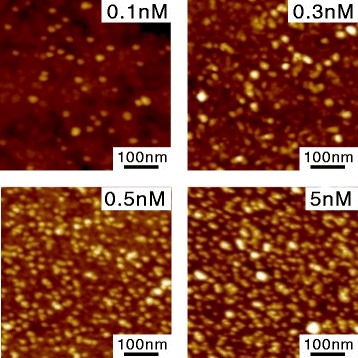
AFM images of QDs on the flat Au surface. The concentrations of QD solution were 0.1, 0.3, 0.5, and 5 nM

In the AFM images of the ultra-flat surfaces that were immersed in QD solutions, many globules can be clearly observed (Fig. 3). These globules were approximately 8 nm in height, consistent with the size of the QDs. On the surface that immersed in 0.1 nM QD solution, the number of globules was approximately 60, across a total area of 500×500 nm^2^. The number of globules increased with the concentration of the QD solution: 160 for 0.3 nM, 180 for 0.5 nM, and 480 for 5 nM. This concentration dependence suggests that the globules were indeed the QDs immobilized on the surface.

**Fig. 4:**
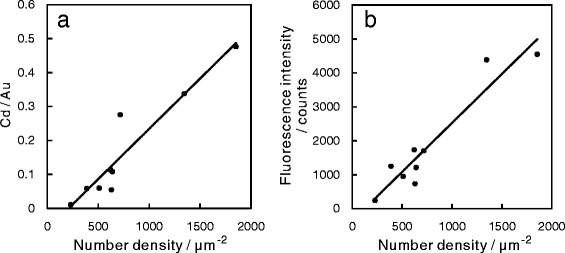
**a** Number density of QDs and atomic ratio of Cd/Au. **b** Number density of QDs and fluorescence intensity

The number density, which was calculated from images of five different areas, was proportional to the atomic ratio of Cd/Au and also supports that the globules were the QDs (Fig. 4 a). The fitting line is projected to cross the *x*-axis, representing the number density, at around 200. This indicates that a number density of 200 *μ* *m* ^−2^ QDs is the limit of detection by XPS measurement. If the number density of QDs having a diameter of 8 nm on an Au surface is 200 *μ* *m* ^−2^, the QDs cover approximately 1% of the surface, which is too small to be analyzed with XPS. In addition, the QDs used in this study includes other elements, such as Se, Zn, S, and C, implying that the actual amount of Cd in the QDs adsorbed at a density of 200 *μ* *m* ^−2^ is less than the detection limit.

The detection limit in the fluorescence measurements was also found to be approximately 200 *μ* *m* ^−2^ in Fig. 4 b. The QDs also showed the blinking behavior, attributed to a fraction of the QDs switching intermittently to the off state (without fluorescence). Moreover, the NA and magnification of the objective lens used in this measurement were relatively small. Considering all these factors, the measured intensity should be smaller than the total of fluorescence actually emitted from the QDs on the surface, making a density as low as 200 QDs *μ* *m* ^−2^ potentially difficult to detect.

### QDs on the Patterned Surface

**Fig. 5:**
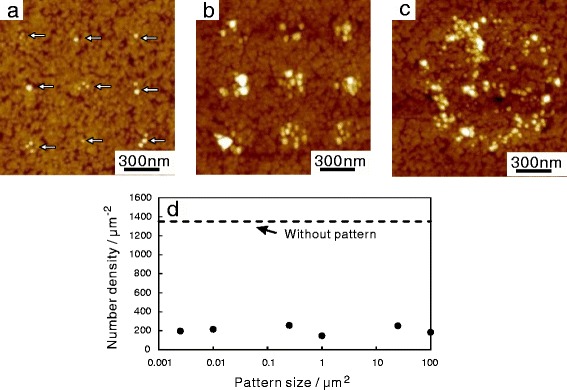
AFM images of QDs on the *dot patterns*. The designed *dot sizes* were 50 nm (**a**), 100 nm (**b**), and 500 nm (**c**). Immobilized QDs are indicated with *white arrows* in **a**. **d** Number density of QDs on the *dot patterns*. *Dashed line* represents the number density of QDs on the Au surface *without pattern*

QDs were immobilized on the dot patterns that were fabricated by e-beam lithography. Typical AFM images of the QDs on the patterns are shown in Fig. 5. In the patterns with the smallest dots, where the designed shape of each dot was a square 50 nm on each side (Fig. 5 a), between 1 and 3 QDs were observed on each dot, as indicated with arrows in the figure. The mean number of QDs on each dot was 1.5. Thus, even though such a small number of QDs would be difficult to analyze with XPS, they were successfully identified by AFM imaging on the ultra-flat Au surface. The number of QDs on the patterns increased with the increase of the dot size: approximately 6 QDs per dot on the square dots with sides of length 100 nm (Fig. 5 b) and 170 QDs per dot on the square dots with sides of length 500 nm (Fig. 5 c).

The actual areas on which the QDs were immobilized were larger than the designed sizes of the dot, because of an overdose of the electron beam. In Fig. 5 c, for example, although the designed size was 500 nm, QDs were immobilized on a circular area with a diameter of 900 nm. The spatial distribution of QDs on the pattern with 500-nm dot sides was not uniform; rather, the dots were mostly found on the periphery of the circular area. The overdose of the electron beam denatured the resist polymer, as well as expanding the area on which the SAM formed. The denatured polymer fractions remained as residues on the surface and prevented the formation of SAMs and immobilization of QDs, which resulted in the inhomogeneous distribution. In the patterns with smaller dots, no such inhomogeneity in the distribution was not noticeable.

The number density of QDs on the dot patterns did not depend on the pattern size and was approximately one seventh of that of QDs on the surface without pattern (Fig. 5 d dashed line). This decrease in the number density was a natural consequence of the residue of the resist on the surface. The immobilization of QDs could only occur on regions of bare Au surface, appropriate for SAM formation, whereas large areas were covered by the residue of the resist. Therefore, this reduction of the area suitable for immobilization of QDs reduced the amount of QDs on the patterned surfaces. In addition, during the lift-off process, by which the resist was removed by solvent after immobilization of QDs, some disruption may have occurred to the amide binding, SAM, or the polymer covering the QDs. Optimizing the dose of the electron beam should improve the yields of immobilized QDs on a patterned surface.

## Conclusions

QDs were immobilized on an ultra-flat Au surface by amide binding between the amino-terminated surface and the carboxyl groups on the QDs. The amount of QDs on the surface, which was analyzed by XPS and fluorescence microscopy, agreed with the Langmuir adsorption isotherm. The QDs on the ultra-flat Au surface were clearly identified by AFM, and their number density was proportional to the amount of QDs as calculated by XPS and fluorescence microscopy.

Furthermore, the use of AFM made it possible to distinguish a few QDs, or even a single QD, immobilized in a pattern on the ultra-flat Au surface. Such small clusters of QDs are difficult to analyze by XPS. The number density of QDs on the patterned surface was less than that on a surface without a pattern. This reduction was attributed to the residue of the resist used in the patterning process and to damages to the SAM and/or amide binding during the lift-off process. These results provide invaluable information on the control of number and position in immobilization of QDs.

## Abbreviations

AFM: Atomic force microscopy
EMCCD: Electron-multiplying charge coupled device
NA: Numerical aperture
QD: Quantum dot
SAM: Self-assembled monolayer
SEM: Scanning electron microscopy
XPS: X-ray photoelectron spectroscopy
